# *Drusus sharrensis* sp. n. (Trichoptera, Limnephilidae), a new species from Sharr National Park in Kosovo, with molecular and ecological notes

**DOI:** 10.3897/zookeys.559.6350

**Published:** 2016-02-03

**Authors:** Halil Ibrahimi, Simon Vitecek, Ana Previšić, Mladen Kučinić, Johann Waringer, Wolfram Graf, Miklós Balint, Lujza Keresztes, Steffen U. Pauls

**Affiliations:** 1 Department of Biology, Faculty of Mathematical and Natural Sciences, University of Prishtina “Hasan Prishtina”, “Mother Theresa” street p.n. 10000 Prishtina, Republic of Kosovo; 2 Department of Limnology and Bio-Oceanography, Faculty of Life Sciences, University of Vienna, Althanstrasse 14, A-1090 Vienna, Austria; 3 Department of Biology, Faculty of Science, University of Zagreb, Rooseveltov trg 6, 10000 Zagreb, Croatia; 4 Institute of Hydrobiology and Aquatic Ecology Management, University of Natural Resources and Life Sciences, Max-Emanuel-Strasse 17, A-1180 Vienna, Austria; 5 Senckenberg Biodiversity and Climate Research Centre (BiK-F), Senckenberganlage 25, 60388 Frankfurt a. M., Germany; 6 Hungarian Department of Biology and Ecology, Babeş-Bolyai University, Cluj-Napoca, Romania

**Keywords:** Caddisfly, Drusinae, Europe, Sharr Mountains, taxonomy, freshwater biodiversity

## Abstract

In this paper we describe *Drusus sharrensis* **sp. n.**, from the Sharr Mountains in Kosovo. Males of the new species are morphologically most similar to *Drusus krusniki* Malicky, 1981, *Drusus kerek* Oláh, 2011 and *Drusus juliae* Oláh, 2011 but differ mainly in exhibiting (1) a differently shaped spinose area on tergite VIII; (2) intermediate appendages anteriorly curved in lateral view with broad tips in dorsal view; (3) inferior appendages with a distinct dorsal protrusion in the proximal half. Females of the new species are morphologically most similar to *Drusus krusniki*, *Drusus kerek*, *Drusus juliae*, and *Drusus plicatus* Radovanovic, 1942 but mainly differ in (1) segment X that is longer than the supragenital plate with distinctly pointed tips; (2) supragenital plate quadrangular with a distinct round dorsal protrusion; (3) a vulvar scale with a small median lobe. Results of phylogenetic species delimitation support monophyly of *Drusus sharrensis* sp. n. and recover it as sister to a clade comprising (*Drusus pelasgus* Oláh, 2010 + *Drusus juliae* + *Drusus arbanios* Oláh, 2010 + *Drusus plicatus* + (*Drusus dacothracus* Oláh, 2010 + *Drusus illyricus* Oláh, 2010)). The new species is a micro-endemic of the Sharr Mountains, a main biodiversity hotspot in the Balkan Peninsula. Main threats to the aquatic ecosystems of this part of the Balkan Peninsula are discussed.

## Introduction

The genus *Drusus* Stephens contains the greatest number of species within the Drusinae. Members of the genus mostly inhabit the European continent with a few additional species known from Asia Minor. Within Europe, the Balkan Peninsula is recognized as one of the most important diversity hotspots of this genus (e.g., Kumanski 1973, Kučinić et al. 2011, Malicky 2004, Oláh 2010, Oláh and Kovács 2013, Previšić et al. 2014a, 2014b, Sipahiler 1999, 2002, Vitecek et al. 2015b, 2015c, Waringer et al. 2015).

The Sharr Mountains represent the border area of three countries, i.e., the Republic of Kosovo, Macedonia, and a small portion extending into north-eastern Albania. This region is characterized by substantial forest ecosystems, diverse geomorphological and hydrological features, and high numbers of endemic and relict species. The name of this mountain range appears in antiquity as “Scardus” “Scordus” or “Scodrus” (Smith 1870) and is reflected in several scientific names, mostly of plants (e.g. *scardicus*, *scardicum*, *scardica*, *scardicolum*, *schariensis*) (Anonymous 2010). The Sharr National Park covers five municipalities (Dragash, Prizren, Suharekë, Shtërpce and Kaçanik) in Kosovo with 36% of its total area covered by forest.

Due to the lack of systematic inventories, biodiversity data for the Sharr Mountains in all three countries are incomplete and are mostly limited to several plant groups, or large mammals. Data for reptiles, amphibians, small mammals, fish, and particularly insects are scarce and outdated (Hajredini et al. 2013). Among insects, the butterfly fauna of the Sharr Mountains is best known, with 147 species registered (Jakšić 1998).

In an ongoing project on the caddisfly fauna of Kosovo (e.g., Ibrahimi et al. 2012a, 2012b, 2013), we sampled caddisfly communities in the Sharr National Park. In this contribution we describe a new *Drusus* species from the Sharr Mountains.

## Materials and methods

We collected adult caddisflies with entomological nets and handpicking from the riparian vegetation near the streams, and nocturnal light trapping in the vicinity of the streams. Nocturnal light trapping followed Malicky’s (2004) protocols. All collected specimens were stored directly in 96% ethanol. The collected material is deposited in the Department of Biology, Faculty of Mathematics and Natural Sciences, University of Prishtina “Hasan Prishtina”, Prishtinë, Republic of Kosovo (DBFMNUP), Croatian Natural History Museum, Zagreb, Croatia (coll. Kučinić-Trichoptera) (CNHM), and Biologiezentrum des Oberösterreichischen Landesmuseums, Linz, Austria (BDOL).

Morphological characteristics of male terminalia were examined in cleared specimens. Specimens were cleared using either the Qiagen Blood and Tissue Kit for DNA-extraction according to the manufacturer’s recommendation and subsequent KOH-treatment (Böhm et al. 2011), or KOH-treatment. Nomenclature of male terminalia follows Nielsen (1957, for *Limnephilus flavicornis* Fabricius) using the simplifying terms “superior appendages” for the lateral processes of segment X (cerci *sensu* Snodgrass 1935), and “intermediate appendages” for the sclerite and the anterior process of segment X (paraproct *sensu* Snodgrass 1935). Illustrations were prepared according to Thomson and Holzenthal (2010) in which pencil drawings made with a camera lucida were digitized, edited, and inked in Adobe Illustrator (v. 16.0.4, Adobe Systems Inc.).

Whole genomic DNA was extracted from the abdomen or the thorax of adult or larval specimens using the DNEasy Blood and Tissue Kit (Qiagen) according to the manufacturer’s protocol. Standard PCR procedures and primers were used to amplify three mitochondrial gene regions (mtCOI5-P, mtCOI3-P, 16SrDNA) and three nuclear gene regions (CADH, WG, 28SnrDNA) (Table 1). PCR reactions were set up in 10µl reactions. Unpurified PCR products were sequenced on an ABI 3177XL capillary sequencer at the Biodiversität und Klima-Forschungszentrum (BiK-F, Frankfurt am Main, Germany) using the PCR primers and two additional internal primers for 28SrDNA (D2UP-4 and D2DN-B, Zhou et al. 2007).

**Table 1.** PCR primers and PCR cycling conditions.

Fragment	Primers & Primer Concentration		PCR Cycling conditions	Taq Kit	Additional Reagents
mtCOI5-P	HCO2198 & LCO1490 (Folmer et al. 1994)	0.25 µM	5'95°C, 5 x (30"95°C, 1'44°C, 1'72°C), 15x (30"95°C, 30"48°C, 1'72°C), 20 x (30"95°C, 30"50°C, 1' + (10'’ * n) 72°C)	peqGOLDHotTaq	-
mtCOI3-P	Jerry & S20 (Pauls et al. 2006)	0.25 µM	5'95°C, 35 x (45"95°C, 30"45°C, 45"72°C), 5'72°C	peqGOLDHotTaq	-
16SrDNA	Lepto-F &Lepto-R (Malm and Johanson 2008)	0.75 µM	3'95°C, 35 x (30"95°C, 30"52°C, 40"72°C), 5'72°C	peqGOLDHotTaq	4 mg BSA
WG	WGbDrrev (5'-accctctcccgcarcacttgag) &WGbDrfwd (5'-cttgctggatgcgtctgcc)^1^	0.5 µM	5'95°C, 35 x (45"95°C, 45"60°C, 90"72°C), 7'72°C	QiagenHotstarTaq plus Master mix	-
CADH	1028r-ino &743nF-ino (Johanson and Malm 2010)	0.25 µM	5'95°C, 35 x (45"95°C, 30"50°C, 45"72°C), 5'72°C	peqGOLDHotTaq	-
28SnrDNA	D1-3up1 (5'-CGAGTAGCGGCGAGCGAACGGA) & D3-TRIC-DN (5'-ATTCCCCTGACTTCGACCTGA)^2^	0.25µM	3'95°C, 35 x (45"95°C, 45"60°C, 60"72°C), 5'72°C	peqGOLDHotTaq	2 mg BSA, 5% DMSO

1: unpublished primer sequence by M. Bálint 2: unpublished primer sequence by K. Kjer

Sequences were edited in Geneious R6 (http://www.geneious.com, Kearse et al. 2012) and aligned using MAFFT v7 (Katoh and Standley 2013) as implemented in Geneious R6. Nucleotide substitution models for each partition were selected according to the Bayesian Information Criterion in the model test module of Mega v5.1 (Tamura et al. 2007) (Table 2). For phylogenetic analysis, the 16SrDNA and 28SnrDNA fragments were not partitioned.

**Table 2.** Substitution models used in phylogenetic analysis.

Fragment	unpartitioned	codon position 1	codon position 2	codon position 3
mtCOI5-P	GTR+G+I	TN93+G	TN93+G	HKY
mtCOI3-P	GTR+G+I	TN93+G+I	K2+G	HKY
16SrDNA	T92+G	-	-	-
WG	T92+G	T92	JC+G	JC
CADH	T92+G+I	HKY+G	TN93	T92
28SnrDNA	T92+G+I	-	-	-

To examine species delineation and association of morphologically similar species of Western Balkan Drusinae, we inferred a phylogeny using all available sequences of the new species (Table 3). As outgroup taxa we used *Drusus discolor* (Rambur, 1842) (Limnephilidae: Drusinae), *Anisogamus waringeri* Graf & Vitecek, 2015 and *Melampophylax austriacus* Malicky, 1990 (Limnephilidae: Stenophylacini) (Table 3).

**Table 3.** Collection data of specimens and length of partial gene sequences used in phylogenetic inference. Abbreviations: Speciment ID, unique study-specific specimen identifier; BOLD ID, BOLD process ID – a unique Barcode of Life Database-specific specimen identifier. Numbers in square parentheses after fragment length indicate number of missing positions. Collectors: AC – Andela Ćukusić, AP – Ana Previšić, BS – Boštjan Surina, DD – Dejan Dmitrović, GS – Goran Šukalo, HI – Halil Ibrahimi, IM – Iva Mihoci, MK – Mladen Kučinić, VK – Vladimir Krpać, WG – Wolfram Graf.

Specimen ID	BOLD ID	28SnrDNA	COI-5P	CADH	COI-3P	16Sr-DNA	Wnt1	Collectors	Coll. date	Latitude (N)	Longitude (E)	Elevation	Taxon
fAns0101L	SPDRU147-14	1038[0n]	658[0n]	848[0n]	541[0n]	360[0n]	0	WG	09.vi.2013	42,4851	2,4134	1888	*Anisogamus waringeri*
fDar0106M	SPDRU163-14	923[84n]	658[0n]	848[0n]	541[0n]	360[0n]	346[0n]	MK, AC	02.vi.2013	40°31.614'	20°25.021'	1920	*Drusus arbanios*
fDar0107M	SPDRU164-14	1040[0n]	658[0n]	848[0n]	541[0n]	360[0n]	346[0n]	MK, AC	02.vi.2013	40°31.614'	20°25.021'	1920	*Drusus arbanios*
fDda0204M	SPDRU227-14	1038[0n]	658[0n]	0	541[0n]	360[0n]	346[0n]	MK, HI, IM, AC	07.vi.2013	41°38.792'	20°11.390'	980	*Drusus dacothracus*
fDda0208M	SPDRU230-14	1036[2n]	658[0n]	848[0n]	541[0n]	360[0n]	346[0n]	MK, HI, IM, AC	07.vi.2013	41°38.792'	20°11.390'	980	*Drusus dacothracus*
fDdd0801M	SPDRU231-14	1038[0n]	658[0n]	848[0n]	541[0n]	362[0n]	346[0n]	AP	10.vii.2013	42,6859	19,7364	960	*Drusus discolor*
fDdd0802F	SPDRU232-14	1038[0n]	658[0n]	848[0n]	541[0n]	362[0n]	346[0n]	AP	10.vii.2013	42,6859	19,7364	960	*Drusus discolor*
fDds0110M	SPDRU243-14	1038[0n]	658[0n]	848[0n]	474[0n]	360[0n]	346[0n]	MK, VK, AC	29.v.2013				*Drusus discophorus*
fDds0111M	SPDRU244-14	1038[0n]	658[0n]	848[0n]	0	360[0n]	346[0n]	MK, VK, AC	29.v.2013				*Drusus discophorus*
fDil0109M	SPDRU268-14	1038[0n]	658[0n]	847[1n]	541[0n]	360[0n]	346[0n]	MK, AC	06.vi.2013	41,5358	20,2279	1830	*Drusus illyricus*
fDju0103M	SPDRU277-14	1038[0n]	658[0n]	848[0n]	541[0n]	362[0n]	346[0n]	MK, HI, IM, AC	04.vi.2013	41°51.848'	20°07.088'	1175	*Drusus juliae*
fDju0104M	SPDRU278-14	1038[0n]	658[0n]	848[0n]	541[0n]	362[0n]	346[0n]	MK, HI, IM, AC	04.vi.2013	41°51.848'	20°07.088'	1175	*Drusus juliae*
fDke0105M	SPDRU280-14	1038[0n]	658[0n]	847[1n]	541[0n]	362[0n]	346[0n]	MK, HI	13.ix.2013	42°31.326'	20°05.919'	2010	*Drusus kerek*
fDke0106M	SPDRU281-14	1036[1n]	658[0n]	848[0n]	541[0n]	362[0n]	346[0n]	MK, HI	13.ix.2013	42°31.326'	20°05.919'	2010	*Drusus kerek*
fDkr0101M	SPDRU294-14	1037[1n]	658[0n]	848[0n]	541[0n]	362[0n]	346[0n]	WG	30.v.2009	42,6438	19,8692		*Drusus krusniki*
fDkr0102M	SPDRU295-14	0	658[0n]	0	541[0n]	362[0n]	346[0n]	WG	30.v.2009	42,6438	19,8692		*Drusus krusniki*
fDpc0106M	SPDRU330-14	1038[0n]	658[0n]	847[1n]	0	360[0n]	346[0n]	MK, VK	31.v.2012	41,7902	20,6348	1279	*Drusus plicatus*
fDpe0105M	SPDRU334-14	1038[0n]	658[0n]	848[0n]	541[0n]	360[0n]	346[0n]	MK, HI, IM, AC	28.vii.2012	41°48.143'	20°33.285'	2300	*Drusus pelasgus*
fDpe0106F	SPDRU335-14	1038[0n]	658[0n]	845[3n]	541[0n]	327[0n]	346[0n]	MK, HI, IM, AC	28.vii.2012	41°48.143'	20°33.285'	2300	*Drusus pelasgus*
fMelaus0101M	SPDRU496-14	1038[0n]	658[0n]	842[6n]	541[0n]	361[0n]	0	WG	20.x.2013	46,8106	14,9931		*Melampophylax austriacus*
fMelaus0102F	SPDRU497-14	1038[0n]	658[0n]	843[5n]	0	361[0n]	0	WG	20.x.2013	46,8106	14,9931		*Melampophylax austriacus*
fDsp4403F	SPDRU545-15	1002[0n]	658[0n]	850[0n]	541[0n]	360[0n]	345[0n]	HI	21.v.2014	42,17228	20,98823	1558	*Drusus sharrensis*sp. n.
fDsp4402M	SPDRU544-15	1002[0n]	454[0n]	848[2n]	541[0n]	360[0n]	345[0n]	HI	21.v.2014	42,17228	20,98823	1558	*Drusus sharrensis*sp. n.
fDsp4401M	SPDRU543-15	1002[0n]	658[0n]	849[1n]	541[0n]	360[0n]	345[0n]	HI	21.v.2014	42,17228	20,98823	1558	*Drusus sharrensis*sp. n.
fDsp4501M	SPDRU546-15	1038[0n]	658[0n]	0	542[0n]	362[0n]	345[0n]	DD, GS	01.x.2014	44,5489	17,3927	393	*Drusus crenophylax*
fDsp4502L	SPDRU547-15	1037[0n]	658[0n]	850[0n]	542[0n]	362[0n]	345[0n]	DD, GS	19.x.2014	44,55	17,393	456	*Drusus crenophylax*

To assess potential conflicts or incongruence among gene fragments, B/MCMCMC single gene analyses were conducted in MrBayes 3.2 (Ronquist et al. 2012), implementing the respective substitution models. Four parallel runs with twelve chains each were performed (10×10^6^ generations, sampling every 5000^th^ generation). Stationary distribution of runs in the same optimal tree space was assumed if the average standard deviation of split frequencies reached values below 0.01. Additionally, MrBayes parameter files were examined in Tracer v1.8 (Rambaut et al. 2014) to assess if runs had reached a stationary phase and converged on model parameters. For each partition, a majority clade credibility tree was estimated based on trees sampled by MrBayes after discarding the first 600 trees of each run as burn-in. Datasets were concatenated as no conflicts among data sets were found, indicating homogeneity of phylogenetic signal from each partition.

Bayesian inference of the concatenated dataset (mtCOI5-P + mtCOI3-P + 16SrDNA + CADH + WG + 28SnrDNA) was performed in MrBayes 3.2, implementing the respective substitution models. Four parallel runs with twelve chains each were carried out (10×10^6^ generations, sampling every 5000^th^ generation). Analytical parameters were examined as stated above. A majority clade credibility tree was estimated based on trees sampled by MrBayes after discarding the first 600 trees of each run as burn-in.

## Results

### Species description

#### Drusus sharrensis

Taxon classificationAnimaliaORDOFAMILIA

Ibrahimi, Vitecek & Previšić sp. n.

http://zoobank.org/0DBB5862-13D4-40FB-98B5-D78288318B1C

##### Material examined.

**Holotype.** 1 male: Republic of Kosovo, Shtërpce Municipality, Sharr Mountains, tributary of the Lepenc River, 2 km above the main road Prizren – Shtërpce, 1558 m, 42.17228°N, 20.98823°E, 21.v.2014, leg. Halil Ibrahimi (DBFMNUP). **Paratypes**: same collection and locality data as holotype, 6 males, 3 females (DBFMNUP), 2 males, 1 female (CNHM), 2 males, 1 female (BDOL); same except 8.v.2014, 2 males, 1 female (CNHM); same except 15.vi.2013, leg. Halil Ibrahimi and Joachim Milbradt, 3 males (DBFMNUP); Shtërpce Municipality, Sharr Mountains, small spring, a branch of the Lepenc River 50 meters above the main road Prizren – Shtërpce, 1410 m, 42.17506°N, 20.97593°E, 08.vi.2010, leg. Halil Ibrahimi, 2 males (DBFMNUP); Shtërpce Municipality, Sharr Mountains, Lepenc River on the main road Prizren – Shtërpce, 1465 m, 42.1813°N, 20.9781°E, 18.v.2010, leg. Halil Ibrahimi, 2 males (DBFMNUP); Prizren Municipality, Sharr Mountains, Lumbardhi i Prizrenit River, Prevallë village 1664 m, 42.161°N, 20.99533°E, 08.vi.2009, leg. Halil Ibrahimi, 1 male (DBFMNUP); Prizren Municipality, Sharr Mountains, first small lake above Prevallë village, 2142 m, 42.152402°N, 20.995024°E, 18.ix.2010, leg. Halil Ibrahimi, 3 males, 1 female (DBFMNUP).

##### Distribution.

Republic of Kosovo, Sharr Mountains.

##### Diagnosis.

Males of the new species are most similar to *Drusus krusniki*, *Drusus kerek* and *Drusus juliae* but differ in exhibiting (1) a dorsally distinctly indented tergite VIII; (2) a narrow, laterally suboval, caudally protruding spinose area of tergite VIII that is medially indented; (3) anteriorly curved intermediate appendages with broad tips; (4) inferior appendages with a distinct dorsal protrusion in the proximal half; (5) parameres with 3 distinct medial spines. *Drusus krusniki* males have (1) a flat, caudally depressed tergite VIII lacking a distinct indentation; (2) a laterally broad, subtriangular, almost straight spinose area of tergite VIII lacking an indentation; (3) intermediate appendages straight, with narrow tips, in lateral view protruding somewhat dorsocaudad; (4) inferior appendages with a slight dorsal protrusion in the proximal half; (5) parameres with a single, dorsal spine in the posterior half and several medial small spines. *Drusus kerek* males have (1) a flat tergite VIII lacking a distinct indentation; (2) a laterally narrow, suboval, almost straight spinose area of tergite VIII lacking an indentation; (3) straight intermediate appendages, with narrow tips; (4) inferior appendages subconical, curved dorsad; (5) parameres with 3 distinct medial spines. *Drusus juliae* males have (1) a rounded tergite VIII lacking a distinct indentation; (2) broad, subtriangular, spinose area of tergite VIII lacking an indentation, lateral parts of spinose area protrude caudad; (3) straight intermediate appendages, tips in dorsal view narrow, in lateral view somewhat pointed posteriad; (4) inferior appendages subconical, curved dorsad; (5) parameres with a single, dorsal spine in the posterior third and several medial small recumbent spines.

Females of the new species are most similar to *Drusus krusniki*, *Drusus kerek*, *Drusus juliae*, and *Drusus plicatus* but differ in exhibiting (1) segment X longer than the supragenital plate with distinctly pointed tips, distally tall in lateral view, caudal margin shallowly concave in dorsal view; (2) a quadrangular supragenital plate with a distinct round dorsal protrusion; (3) a vulvar scale with a small median lobe. *Drusus krusniki* females have a more-slender segment X that is shorter than the supragenital plate in dorsal view and has round tips and a deeply concave caudal margin. *Drusus kerek* females have a ventrally curved segment X shorter than the supragenital plate, a dorsally irregularly rounded supragenital plate, and a vulvar scale lacking the median lobe. *Drusus juliae* females have round tips of segment X and lack a distinct dorsal protrusion of the supragenital plate. *Drusus plicatus* females have a more-slender segment X that is shorter than the supragenital plate in dorsal view and has round tips and a deeply concave caudal margin, and a rounded supragenital plate in ventral view that lacks a distinct dorsal protrusion in lateral and caudal views.

##### Description.

*General appearance*. Habitus dark; sclerites and tergites dark brown; cephalic and thoracic setal areas pale; cephalic, thoracic and abdominal setae blond; legs brown to fawn, proximally darker; haustellum and intersegmental integument pale, whitish. Wings dark brown with dark setae. Male maxillary palp 3-segmented. Forewing length 11–12.5 mm, spur formula 1–3–3 in males; forewing length 11.5–13 mm, spur formula 1–3–3 in females.

**Figure 1.**
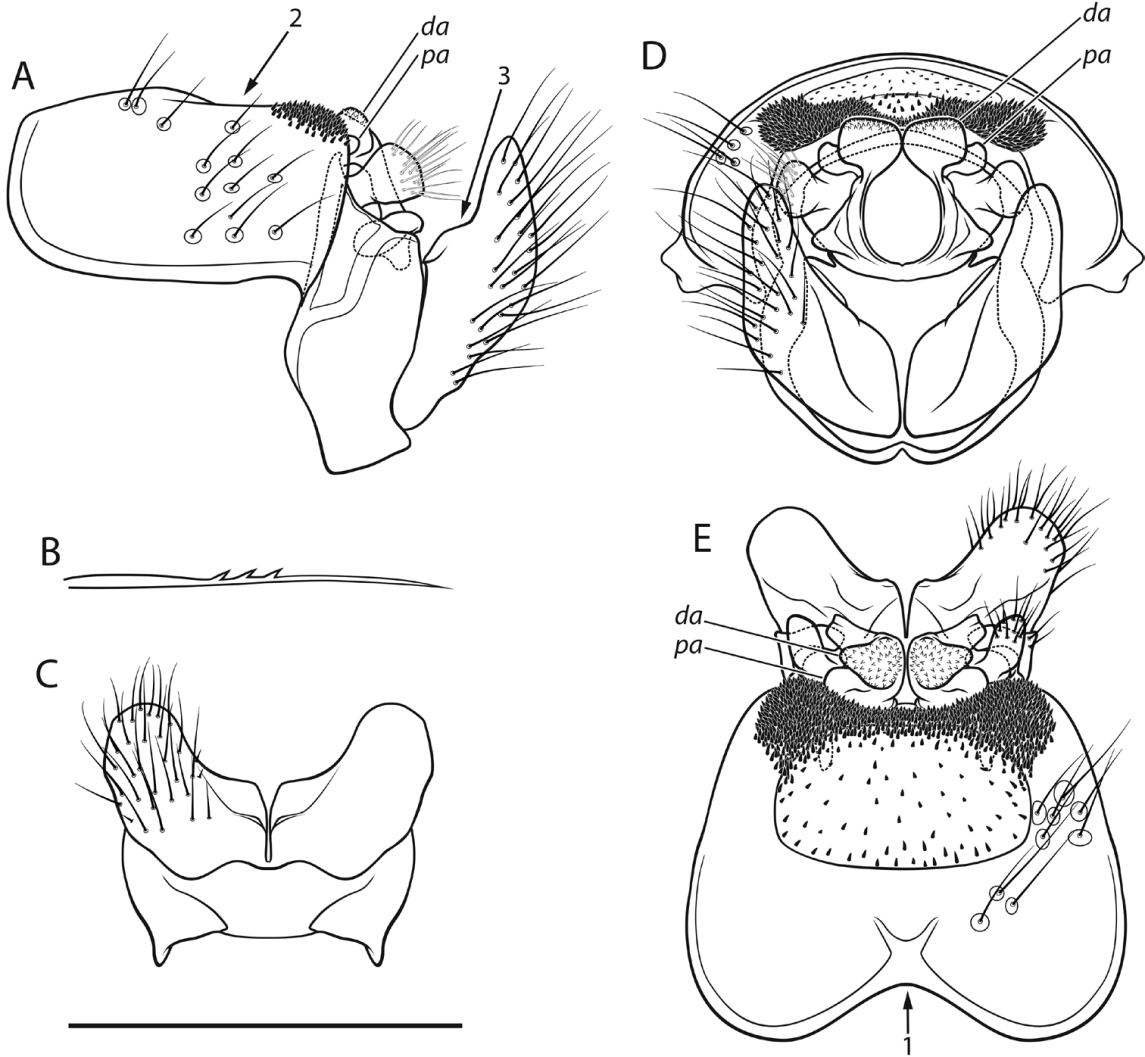
Male genitalia of *Drusus sharrensis* sp. n.: **A** left lateral view **B** paramere left lateral view **C** ventral view **D** caudal view **E** dorsal view. Small letters and numbers indicate structures referred to in the description. Scale bar 1 mm. Illustrations by S. Vitecek.

**Figure 2.**
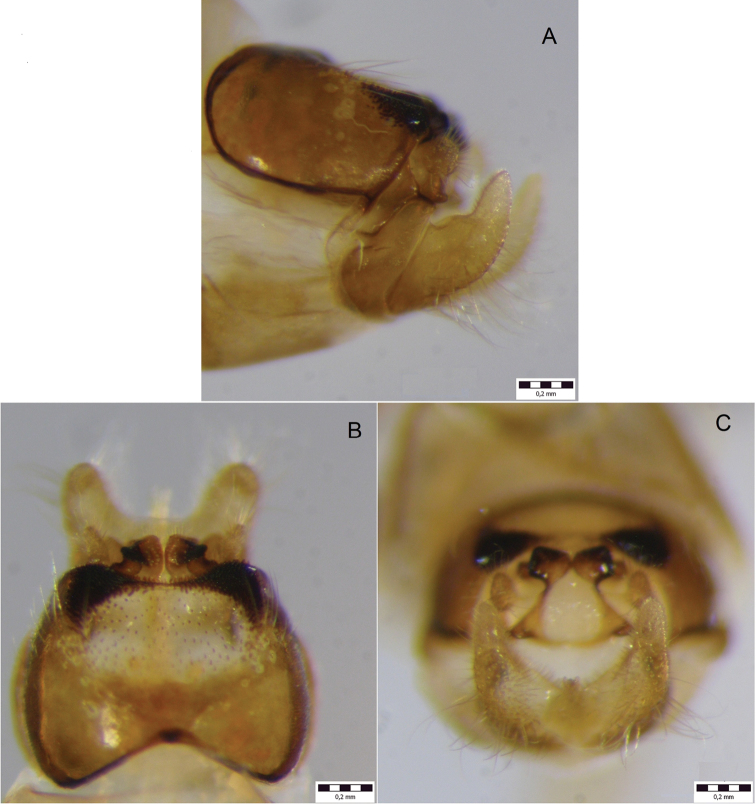
Male genitalia of *Drusus sharrensis* sp. n. **A** left lateral view **B** dorsal view **C** caudal view.

*Male genitalia* (Fig. 1A–E; Fig. 2A–C). Tergite VIII dark brown, in dorsal view distinctly incised anteriorly (*arrow 1*, Fig. 1); setation concentrated laterally; spinose area divided into two suboval laterocaudal lobes medially connected by band of spines, embracing distinct medial, indented, weakly sclerotized (translucent in cleared specimens) oval area with few spines (*arrow 2*, Fig. 1). Ninth abdominal segment in caudal view widest ventrally; in lateral view with rounded apical protrusion at the base of the intermediate appendages, medially widest mid-height, apical margin ventrally concave with slight ventral protrusion embracing ventral base of inferior appendages (gonopods *sensu* Snodgrass 1935). Superior appendages in lateral view short, proximally constricted, suboval, ventroposterially somewhat pointed. Intermediate appendages in lateral view dorsally curved anterad, dorsal tip of each with proximal and distal aspect separated by distinct indentation: proximal aspect (*pa*, Fig. 1) rounded, flat, distal aspect (*da*, Fig. 1) curving anterad, rough; tips in dorsal view approximately parallel, proximal section rounded, extending laterad, the distal end subtriangular with rounded corners, medially somewhat dilated, rough; in caudal view approximately trapezoidal, proximal tips wider than distal tips and slightly pointed dorsad, distal tips rounded. Inferior appendages in lateral view curved dorsad, proximally with distinct dorsal protrusion (*arrow 3*, Fig. 1), ventral margin proximally slightly indented; in dorsal and ventral views with subtriangular median lobe separated by longitudinal grooves; in dorsal, ventral, and caudal views proximally laterally protruding, distally approximately straight in dorsoventral plane; in caudal view inferior appendages suboval; in ventral view inferior appendages seemingly medially fused proximally. Parameres simple, with 3 distinct median spines.

**Figure 3.**
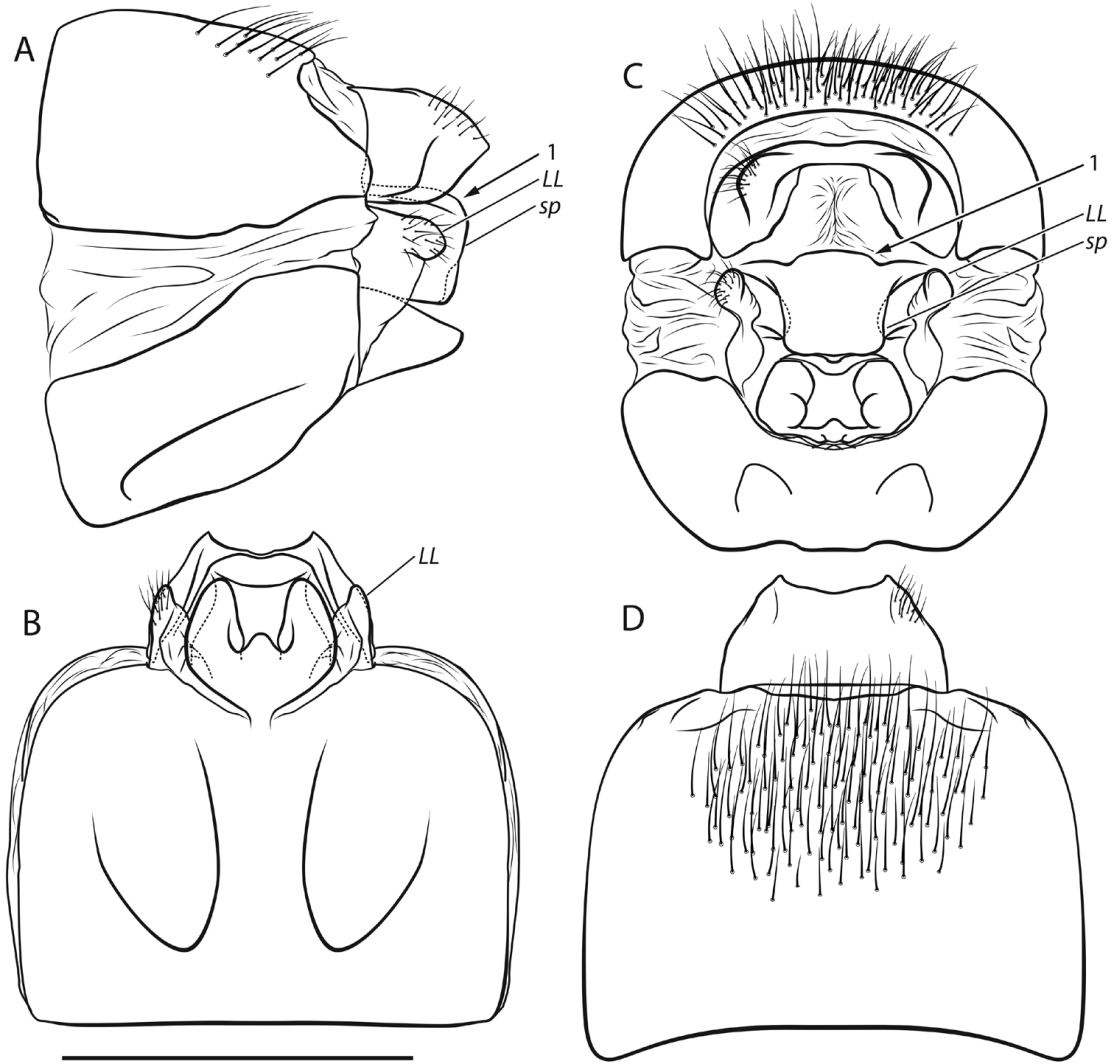
Female genitalia of *Drusus sharrensis* sp. n.: **A** left lateral view **B** ventral view **C** caudal view **D** dorsal view. Scale bar 1 mm. Small letters and numbers indicate structures referred to in the description. Illustrations by S. Vitecek.

**Figure 4.**
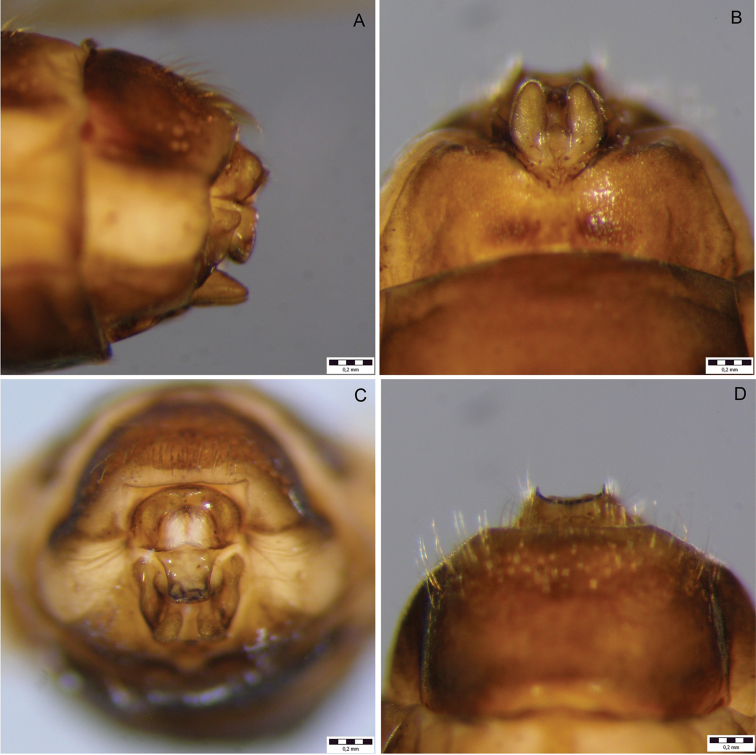
Female genitalia of *Drusus sharrensis* sp. n.. **A** left lateral view **B** ventral view **C** caudal view **D** dorsal view.

*Female genitalia* (Fig. 3A–D; Fig. 4A–D). Segment IX setation abundant, concentrated in caudal half; lateral lobe (LL, Fig. 3) of segment IX membranous, in lateral view right-angled triangular, the assumed adjacent angle about twice as long as the assumed opposite angle with dorsal sclerotized setose lobe protruding caudad; in dorsal and ventral views slender, projecting caudad; in caudal view dorsal sclerotized setose part rounded, well separated from membranous part. Segment X longer than supragenital plate, in lateral view distally higher than proximally with distinct posterior tip; in dorsal view medially widest, caudally tapering, with 2 small round setose lateral protrusions and distinct tips, apical margin irregularly concave; ventrally unsclerotized, open. Supragenital plate (*sp*, Fig. 3) in lateral view quadrangular with distinct, rounded dorsal protrusion (*arrow 1*, Fig. 3), apical margin ventrally slightly protruding; in ventral view quadrangular, medially concave; in caudal view quadrangular, wider dorsally than ventrally, with distinct rounded dorsal protrusion. Vulvar scale in lateral view subtriangular, slightly curved ventrad, longer than supragenital plate; in ventral view separated from sternite IX by proximal constriction, with 3 lobes: 2 lateral lobes, roundly oval, tapering caudad; median lobe short, wider than long.

##### Etymology.

The species epithet *sharrensis*translates to ‘from [the] Sharr [mountains]’, and was formed by appending the Latin suffix ‘-ensis’ to the actual name of the mountain range where the new species is found. Note: In Albanian ‘Sharr’ also refers to the city of Dragash (Kosovo), the municipality of a large proportion of Sharr Mountains.

##### Ecological notes and distribution.

During our field survey in the Sharr Mountains we found *Drusus sharrensis* at five locations within a 20 km perimeter, between 1410 and 2141 m above sea level. The new species was collected from one spring, two spring brooks and two mid-stream locations of the Lumbardhi i Prizrenit and Lepenc rivers. Substrate of streams close to the sampling sites was dominated by meso- to macrolithal. The highest number of specimens was collected at spring brooks surrounded by dense riparian vegetation. The species was mostly collected during the day with entomological nets – only one male specimen was collected by nocturnal light trapping although the weather was suitable and light trapping effort was considerable, indicating a diurnal activity pattern. The species was collected during May, June, July, and September.

##### Results of phylogenetic species delimitation.

**Figure 5.**
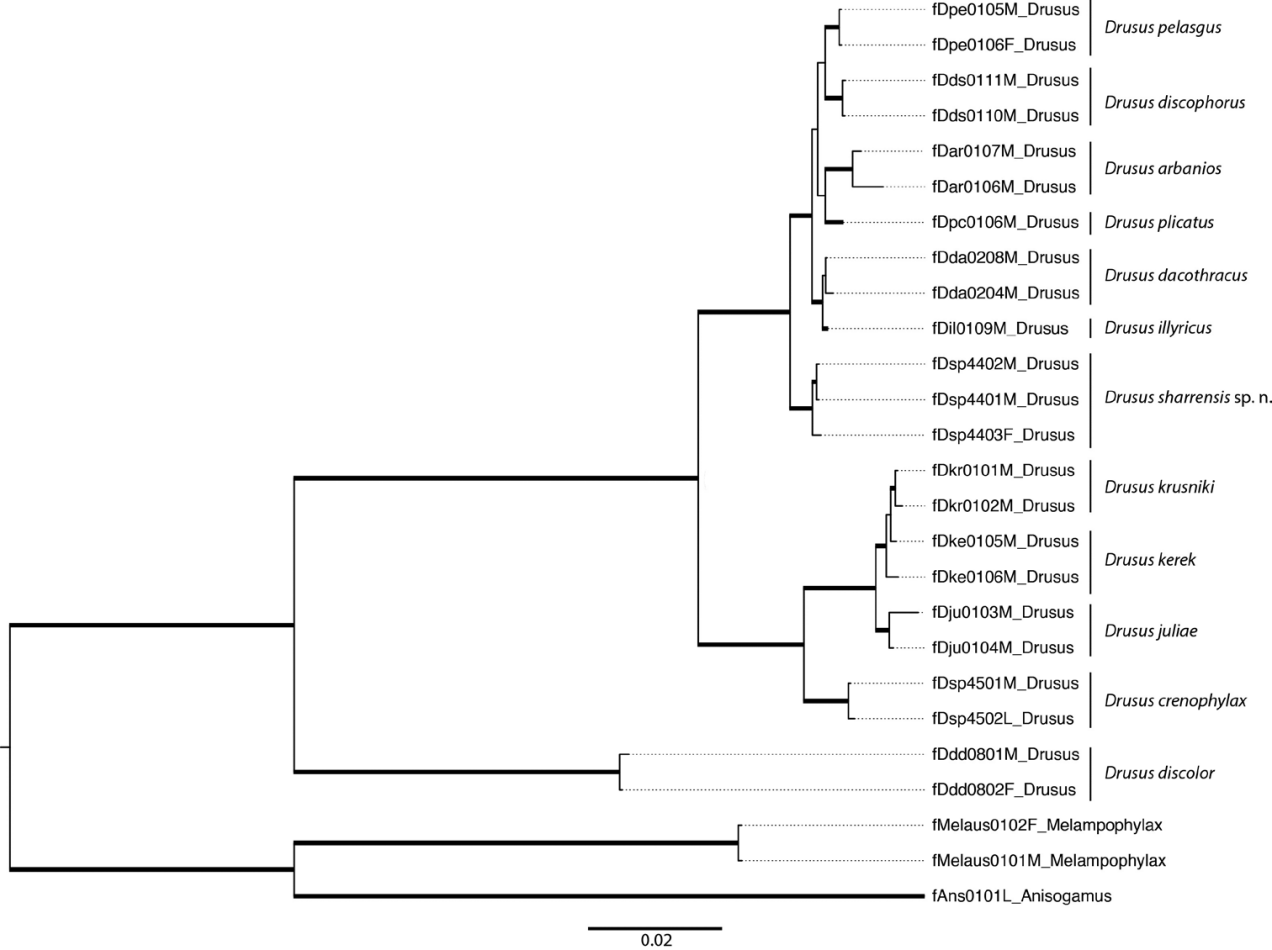
Results of phylogenetic inference. B/MCMC species tree analysis for nine *Drusus* species (26 terminal taxa) based on 3805bp-long sequence from 6 loci (mtCOI5-P, mtCOI3-P, CADH, 16SrDNA, WG, 28SnrDNA).

In a B/MCMCMC phylogeny based on partial sequence data from six loci, monophyly of *Drusus sharrensis* was highly supported (Fig. 5). However, relationships between species were not resolved. The new species *Drusus sharrensis* was recovered, with high support, as sister to a clade comprising (*Drusus pelasgus* + *Drusus discophorus* Radovanovic, 1942 + *Drusus arbanios* + *Drusus plicatus* + (*Drusus dacothracus* + *Drusus illyricus*)). The clade (*Drusus sharrensis* + (*Drusus pelasgus* + *Drusus discophorus* + *Drusus arbanios* + *Drusus plicatus* + (*Drusus dacothracus* + *Drusus illyricus*))) is a derived sister to a clade composed of (((*Drusus krusniki* + *Drusus kerek*) + *Drusus juliae*) + *Drusus crenophylax* Graf & Vitecek, 2015) in which (*Drusus krusniki* + *Drusus kerek*) is recovered as a polytomy, and this relationship was highly supported.

## Discussion

### Systematic position

The combination of the gene fragments mtCOI3-P, 16SrDNA, and WG was previously demonstrated to successfully resolve phylogenetic relationships of Drusinae (Pauls et al. 2008), and was used to delineate species of Western Balkan Drusinae (Previšić et al. 2014a). In the present study, a set of six gene fragments was used to infer phylogenetic relationships of taxa in a Bayesian framework to discriminate species. These genes were successfully employed by Vitecek et al. (2015a) to assess other relationships among Drusinae. Bayesian phylogenetic inference based on the combination of six gene fragments (mtCOI5-P, mtCOI3-P, CADH, 16SrDNA, WG, 28SnrDNA) recovers the new *Drusus* species as monophyletic, and sister to a clade comprising (*Drusus pelasgus* + *Drusus discophorus* + *Drusus arbanios* + *Drusus plicatus* + (*Drusus dacothracus* + *Drusus illyricus*)).

### Ecological notes

Data on the ecology of species closely related to *Drusus sharrensis* are incomplete. From what is known, the emergence pattern of the new species corresponds to that of a related species from Bjeshkët e Nemuna, *Drusus krusniki*. The sex ratio of the new species ranges from 1:2 to1:3 in favour of males at the different sampling locations, similar to sex ratios recorded in *Drusus krusniki* (Ibrahimi et al. 2014b).

### Aquatic insect diversity of Sharr Mountains and main threats

There are currently about 30 stonefly (Dauti 1980) and about 50 caddisfly species (Ibrahimi et al. 2012a, 2012b) known from the Sharr Mountains. Many of these species are rare and/or endemics of the Balkan Peninsula. This number of known aquatic insect species is surely far below the real number inhabiting this range of mountains. The stonefly *Nemoura zwicki* Sivec, 1980 is an endemic species of this mountain range described from a streamlet only a few kilometers away (Sivec 1980) from type locality of the new *Drusus* species. The caddisfly *Limnephilus petri* Marinković-Gospodnetić, 1966 is also an endemic species of the Sharr Mountains (Marinković-Gospodnetić 1966).

The biodiversity of the Sharr Mountains is threatened by illegal logging, water extraction from springs, expansion of touristic activities and several other anthropogenic factors (Flores and Selimi 2013). Several limestone and rock quarries operate in the Sharr Mountains in the vicinity of aquatic ecosystems potentially causing severe siltation. Additionally, recent development of a winter tourism facility at Brezovicë, close to the type locality of *Drusus sharrensis*, may enhance local degradation of terrestrial and, particularly, aquatic ecosystems in the Sharr Mountains through water intake, habitat deterioration, and discharge of sewage effluents. The Brezovica Touristic Centre Development Project was designed by the Government of the Republic of Kosovo with support from the European Union to promote the touristic appeal and thus economic importance of the area. This project will impact a total area of roughly 3,700 ha (Flores and Selimi 2013).

The description of *Drusus sharrensis* is a contribution to the faunistic list of Kosovo caddisflies (Gashi et al. 2015, Malicky 1986, 1999, Marinković-Gospodnetić 1975, 1980, Oláh 2010, Oláh et al. 2013, 2014, Radovanović 1931, Ibrahimi and Gashi 2008, Ibrahimi et al. 2012a, 2012b, 2013, 2014a, 2014b, 2015). Further, the description of the new species highlights the importance of this rapidly changing area to local and regional biodiversity.

## Supplementary Material

XML Treatment for Drusus sharrensis
